# Prognostic Relevance of Cytokine Receptor Expression in Acute Myeloid Leukemia: Interleukin-2 Receptor α-Chain (CD25) Expression Predicts a Poor Prognosis

**DOI:** 10.1371/journal.pone.0128998

**Published:** 2015-09-16

**Authors:** Kazunori Nakase, Kenkichi Kita, Taiichi Kyo, Takanori Ueda, Isao Tanaka, Naoyuki Katayama

**Affiliations:** 1 Cancer Center, Mie University Hospital, Tsu, Japan; 2 Department of Internal Medicine, Japan Baptist Hospital, Kyoto, Japan; 3 Fourth Department of Internal Medicine, Hiroshima Red Cross and Atomic-Bomb Survivors Hospital, Hiroshima, Japan; 4 First Department of Internal Medicine, Fukui University School of Medicine, Fukui, Japan; 5 Department of Internal Medicine, Suzuka Kaisei Hospital, Suzuka, Japan; 6 Department of Hematology and Oncology, Mie University Graduate School of Medicine, Tsu, Japan; European Institute of Oncology, ITALY

## Abstract

A variety of cytokine/cytokine receptor systems affect the biological behavior of acute leukemia cells. However, little is known about the clinical relevance of cytokine receptor expression in acute myeloid leukemia (AML). We quantitatively examined the expression of interleukin-2 receptor α-chain (IL-2Rα, also known as CD25), IL-2Rβ, IL-3Rα, IL-4Rα, IL-5Rα, IL-6Rα, IL-7Rα, the common β-chain (βc), γc, granulocyte-macrophage colony-stimulating factor (GM-CSF)Rα, G-CSFR, c-fms, c-mpl, c-kit, FLT3, and GP130 in leukemia cells from 767 adult patients with AML by flow cytometry and determined their prevalence and clinical significance. All cytokine receptors examined were expressed at varying levels, whereas the levels of IL-3Rα, GM-CSFRα, IL-2Rα, γc, c-kit, and G-CSFR exhibited a wide spectrum of ≥10,000 sites/cell. In terms of their French-American-British classification types, GM-CSFRα and c-fms were preferentially expressed in M4/M5 patients, G-CSF in M3 patients, and IL-2Rα in non-M3 patients. Elevated levels of IL-3Rα, GM-CSFRα, and IL-2Rα correlated with leukocytosis. In patients ≤60 years old, higher levels of these 3 receptors correlated with poor responses to conventional chemotherapy, but only IL-2Rα was associated with a shorter overall survival. By incorporating IL-2Rα status into cytogenetic risk stratification, we could sort out a significantly adverse-risk cohort from the cytogenetically intermediate-risk group. Analyses with various phenotypical risk markers revealed the expression of IL-2Rα as an independent prognostic indicator in patients with intermediate-risk cytogenetics. These findings were not observed in patients >60 years old. Our results indicate that several cytokine receptors were associated with certain cellular and clinical features, but IL-2Rα alone had prognostic value that provides an additional marker to improve current risk evaluation in AML patients ≤60 years old.

## Introduction

Numerous cytokines, including colony-stimulating factors (CSF) and interleukins (IL), are produced and released by a variety of cell types and act as intercellular mediators to maintain homeostasis during hematopoiesis, immune responses, and inflammatory reactions [1,2]. These cytokines are also known to regulate the survival, growth, differentiation, and apoptosis of acute leukemia cells, both in vitro and in vivo [2–4]. Cytokines bind to their cognate receptors on the cell surface to exert their biological effects [1,2]. Accordingly, aberrant or excessive expression of cytokine receptors may be closely associated with the pathological status of patients with acute leukemia.

Previous studies have shown that elevated expression of the IL-3 receptor α-chain (IL-3Rα), a cell surface marker of leukemia stem cells (LSCs), correlates with a poor prognosis of acute myeloid leukemia (AML) [5,6]. Several investigators have demonstrated that the expression of IL-2Rα (CD25) correlates with an adverse outcome in patients with AML [7–10] and acute lymphoblastic leukemia [11,12]. Furthermore, IL-2Rα^+^ AML cells were shown to comprise a population of cell cycle-quiescent and chemotherapy-resistant LSCs [13]. However, the expression patterns of various cytokine receptors other than IL-2Rα and IL-3Rα on AML cells have not been extensively investigated [3,4], and little is known about the detailed prognostic relevance of such receptor expression in AML patients [4].

In this study, we quantitatively assessed the cell surface expression levels of cytokine receptors, including IL-2Rα, IL-2Rβ, IL-3Rα, IL-4Rα, IL-5Rα, IL-6Rα, IL-7Rα, the common β-chain (βc), γc, granulocyte-macrophage CSFRα (GM-CSFRα), G-CSFR, M-CSFR (c-fms), the thrombopoietin receptor (TPO-R, also known as c-mpl), stem cell factor receptor (SCF-R, also known as c-kit), fms-related tyrosine kinase 3 (FLT3), and GP130 in leukemia cells from a large population of adult AML patients by flow cytometry and evaluated their prevalence and prognostic significance.

## Materials and Methods

### Patients and samples

Diagnostic bone marrow (BM) and/or peripheral blood (PB) samples from 767 Japanese patients over 15 years of age who were diagnosed with previously untreated, de novo AML were consecutively collected from collaborating hospitals and examined at the Second Department of Internal Medicine at the Mie University School of Medicine. Physicians (including authors of this manuscript) at collaborating hospitals were responsible for obtaining the BM and PB samples. AML diagnoses and classifications were made according to the French-American-British (FAB) classification system. Using the FAB system, patients were classified as follows: 15 patients as M0, 138 as M1, 246 as M2, 107 as M3, 135 as M4, 91 as M5, 17 as M6, 6 as M7, and 12 as unknown. Patients were treated with 1 of 6 Japanese AML multicenter trial regimens (JALSG-AML87, 89, 92, 95, 97, and B-DOMP). The drugs used for induction therapies were as follows: JALSG-AML87 [14], behenoyl cytarabine (BHAC), daunorubicin (DNR), 6-mercaptopurine (6MP), and prednisolone (PSL), which were administered with or without vincristine (VCR); JALSG-AML89 [15], BHAC or cytarabine (Ara-C), DNR, 6MP, and PSL; JALSG-AML92 [16], BHAC, DNR, 6MP with or without etoposide (ETP); JALSG-AML95 [17], Ara-C and idarubicin (IDR); JALSG-AML97 [18], Ara-C and IDR; or B-DOMP [19], BHAC, DNR, VCR, 6MP, and PSL. Post-remission consolidation therapy was performed with or without maintenance chemotherapies for patients who achieved complete remission (CR) after induction chemotherapy. Neither high nor intermediate doses of Ara-C were used with any patients during post-remission therapy. Because the introduction of all-trans retinoic acid has dramatically improved clinical outcomes in patients with AML-M3, these patients were excluded from prognostic assessments. No statistically significant differences in overall survival (OS) rates were observed between groups treated with different AML protocols (data not shown). CR was defined as the presence of less than 5% leukemia cells in BM aspirate after induction chemotherapy. The patient samples were investigated during 1987 to 2000, and their survival data were evaluated until 2010. All the patients`data were anonymized and de-identified prior to analysis. The institutional review board of the Mie University Hospital approved this study and waived the need for individual informed consent due to the retrospective nature of the study.

### Flow cytometric analysis

Mononuclear cells (MNCs) were separated by Ficoll-Hypaque density gradient centrifugation. Immunostaining was performed by a standard indirect immunofluorescence method, as previously described [7,12]. Monoclonal antibodies (mAbs) were used to detect the following cell surface markers and cytokine receptors: CD4, CD7, CD11c, CD18, CD19, CD34, and CD56 (Becton Dickinson, Mountain View, CA); CD2, CD11b, HLA-DQ, and HLA-DR (Ortho, Raritan, NJ); CD14 and CD33 (Coulter, Hialeah, FL); CD13 (CALTAG Laboratories, Burlingame, CA); IL-3Rα (N3A) and βc (5A5) (kindly provided by Dr. T. Kitamura, Tokyo University, Japan); IL-2Rα (anti-Tac) (Dr. T. Uchiyama, Kyoto University, Japan); IL-2Rβ (Mikβ1) (Dr. M. Tsudo, Osaka Red Cross Hospital, Japan); GM-CSFRα, IL-4Rα, IL-7Rα, and c-mpl (Genzyme, Cambridge, MA); c-kit, IL-6Rα, and GP130 (Serotec, Oxford, UK); IL-5Rα, G-CSFR, and γc (Pharmingen, San Diego, CA); c-fms (Santa Cruz Biotechnology, Santa Cruz, CA); and FLT3 (Immunotech, Marseille, France). Regarding anti-Tac, it was firstly developed mAb against IL-2Rα [20], and has been used to detect IL-2Rα in various hematologic malignancies in many studies [7,12,21,22].

A fluorescein isothiocyanate-conjugated goat anti-mouse IgG F(ab)_2_ (GAM-FITC; Coulter) was used as the secondary antibody. Whole mouse Igs, IgG1 and IgG2 (Chemicon, Temecula, CA) were used as the controls. Double immunostaining was performed to determine whether myeloid antigen and IL-2Rα were expressed on the same or different cells. Briefly, cells were first stained with the anti-Tac mAb with indirect fluorescence, and with a phycoerythrin (PE)-conjugated anti-CD13 antibody. Flow cytometric analysis was performed using a Cytron flow cytometer (Ortho). As the cell samples were found to consist of ≥90% leukemia cells in cytospin preparations, samples for the cell surface markers were considered positive if more than 15% of the leukemia cells showed immunofluorescence that was greater than that observed with the negative control. Expression levels of individual cytokine receptors were assessed by measuring the mean fluorescence intensities (MFIs) of cell stained with the related antibodies. Antibody binding capacities (sites/cell) of test samples and control cells were calculated based on MFIs and calibration curves obtained using the DAKO QIFIKIT and TallyCAL software packages (DAKO, Grostrip, Denmark), as described previously [12]. Samples with <200 binding sites/cell were judged as undetectable in this study.

### Cytogenetic analysis

Cytogenetic analyses were performed with BM cells. Chromosomes were classified according to the International System for Human Cytogenetic Nomenclature (ISCN; 1985). Patients were divided into three risk groups of favorable, intermediate, and adverse for cytogenetic abnormalities according to the revised Medical Research Council prognostic classification [23].

### Responsiveness to cytokines

The T- and adherent cell-eliminated MNC fraction was used as the leukemia cell-enriched fraction. Response of leukemia cells to various cytokines was tested by the ^3^H-thymidine incorporation method in short-term liquid cultures as described previously [7]. All experiments were performed in triplicate and data were expressed in terms of the stimulation index (S.I.), which was calculated by dividing the mean value (cpm) of cytokine stimulated cultures with those of control cultures. The cytokines used were recombinant human IL-3 (10 ng/ml), GM-CSF (10 ng/ml), IL-2 (10 U/ml), IL-4 (10 ng/ml), M-CSF (10 ng/ml), and IL-6 (10 ng/ml). IL-6 and G-CSF were kindly provided by Kirin Brewery (Tokyo, Japan), and IL-2 was provided by Takeda Pharmaceutical Co. (Osaka, Japan). IL-3, GM-CSF and IL-4 were purchased from Genzyme, and M-CSF was purchased from R & D systems (Minneapolis, MN).

### Statistical analysis

Differences in the distribution of continuous variables between categories were analyzed using either the Mann—Whitney U test for comparisons between 2 groups or the Kruskal—Wallis test for comparisons between 3 groups. Correlations between 2 categorical variables were evaluated by the Fisher exact test. Analysis of the distribution between 2 continuous variables was performed using the Pearson product-moment correlation test. Patient survival data were calculated by the Kaplan—Meyer method, and differences were evaluated by the log-rank test. Univariate and multivariate analyses for OS were performed using the Cox proportional hazard regression model. OS was measured from the first day of therapy to death. The observation time was censored at the date of stem cell transplantation or the date of the last follow-up. Data were analyzed using STATISTICA software (StatSoft, Tulsa, OK). All *P* values were 2-tailed. Because a large number of significance tests were performed and conventionally significant results (*P* < 0.05) may have been obtained by chance due to the associated increased probability, only *P* values of < 0.01 are reported in this study.

## Results

### Prevalence of cytokine receptor expression in AML

**Fig 1:**
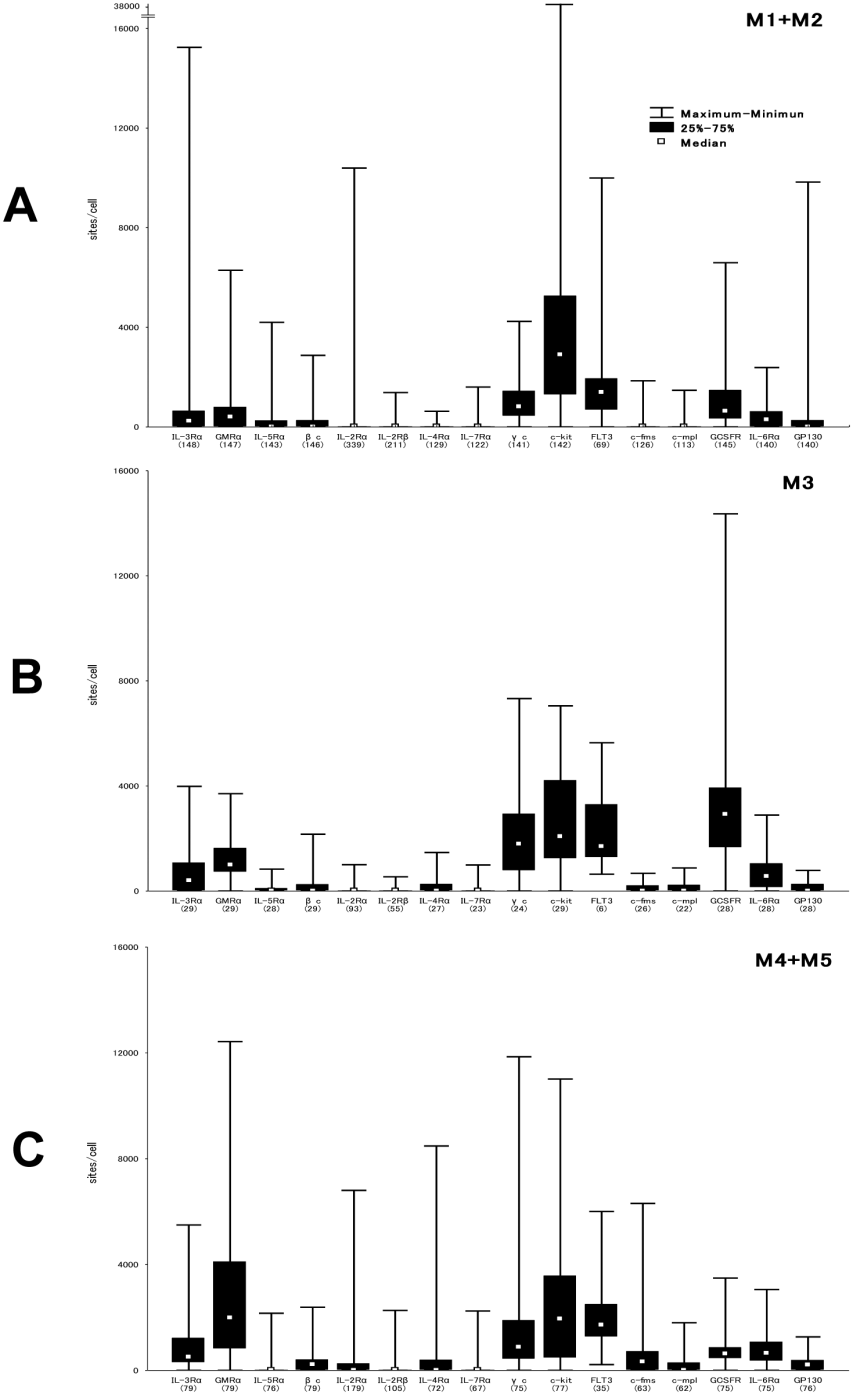
Distribution patterns of cytokine receptor expression based upon FAB subtype groups. Expression levels of cytokine receptors (sites/cell) in patients with AML are shown according to the FAB subtype groups; (A) M1+M2, (B) M3, and (C) M4+M5. The number of patients examined is shown in parenthesis.

Table 1 shows expression levels of IL-3Rα, GM-CSFRα, IL-5Rα, βc, IL-2Rα, IL-2Rβ, IL-4Rα, IL-7Rα, γc, c-kit, FLT3, c-fms, c-mpl, G-CSFR, IL-6Rα, and GP130 observed in leukemia cells from AML patients. All cytokine receptors were expressed in AML cells with variable ranges in terms of sites/cell. IL-3Rα, GM-CSFRα, IL-2Rα, γc, c-kit, and G-CSFR expression levels exhibited a wide spectrum, with observed ranges varying by more than 10,000 sites/cell. Among these receptors, c-kit showed the highest mean level (3,570 sites/cell), median level (2,611 sites/cell), and maximum level (37,385 sites/cell). In contrast, the mean levels of IL-5Rα, IL-2Rβ, IL-4Rα, IL-7Rα, and c-mpl were less than 200 sites/cell, with IL-2Rβ showing the lowest mean level (34 sites/cell). We compared the expression levels of cytokine receptors that showed mean levels >200 sites/cell among 3 FAB subtype groups (M1 + M2; Fig 1A, M3; Fig 1B, M4 + M5; Fig 1C). Statistically significant differences in the expression levels of IL-3Rα (*P* = 0.003), GM-CSFRα (*P* < 0.001), βc (*P* = 0.009), IL-2Rα (*P* < 0.001), c-fms (*P* < 0.001), G-CSFR (*P* < 0.001), and IL-6Rα (*P* < 0.001) were observed among these FAB subtype groups. In particular, GM-CSFRα and c-fms were preferentially expressed in the M4/M5 group, G-CSF in the M3 group, and IL-2Rα in the non-M3 groups. No significant difference was observed in the expression levels of IL-2Rα between the M1+M2 and the M4+M5 groups (*P* = 0.09).

**Table 1:** Expression levels of cytokine receptors in patients with AML.

Cytokine receptor	No.of patients	sites/cell		
		Mean ± SE	Median	Max
**IL-3Rα**	**262**	**840 ± 94**	**377**	**15243**
**GM-CSFRα**	**261**	**1397 ± 111**	**718**	**12428**
**IL-5Rα**	**251**	**186 ± 30**	**0**	**4201**
**βc**	**260**	**203 ±2 5**	**0**	**2879**
**IL-2Rα**	**638**	**309 ± 41**	**0**	**10397**
**IL-2Rβ**	**379**	**34 ± 9**	**0**	**2270**
**IL-4Rα**	**234**	**174 ± 44**	**0**	**8484**
**IL-7Rα**	**217**	**83 ± 18**	**0**	**2248**
**γc**	**244**	**1308 ± 108**	**908**	**17238**
**c-kit**	**252**	**3570 ± 256**	**2611**	**37385**
**FLT3**	**113**	**1840 ± 152**	**1492**	**9999**
**c-fms**	**220**	**235 ± 41**	**0**	**6313**
**c-mpl**	**202**	**118 ± 22**	**0**	**1803**
**G-CSFR**	**252**	**1158 ± 91**	**703**	**14357**
**IL-6Rα**	**249**	**610 ± 49**	**390**	**7191**
**GP130**	**250**	**245 ± 46**	**0**	**9836**

IL-3Rα, interleukin-3 receptor α-chain; GM-CSF, granulocyte-macrophage colony-stimulating factor; βc, common β-chain; G-CSF, granulocyte CSF, SE, standard error.

### Relationship between cytokine receptor expression and clinical and cellular features in AML

We analyzed the relationship between the levels of cytokine receptors that showed the wide range (maximum level ≥ 10,000 sites/cell or mean levels ≥ 1,000 sites/cell) and clinical (age, gender and white blood cell [WBC] count at diagnosis) and cellular features (CD34 expression and cytogenetic risk groups) in patients with AML (Table 2). With regards to the clinical features, elevated levels of γc (*P* = 0.001) and G-CSFR (*P* = 0.006) correlated with a patient age of ≤60 years. WBC counts ≥ 3 ×10^4^/μl was associated with higher expression of IL-3Rα (*P* = 0.00003), GM-CSFRα (*P* = 0.0006), and IL-2Rα (*P* = 0.00006), and with lower expression of c-kit (*P* = 0.0003) and G-CSFR (*P* = 0.00004). Regarding the cellular features, CD34 exhibited a significant correlation with increased levels of IL-2Rα (*P* = 0.0009) and c-kit (*P* = 0.0003), and to decreased levels of GM-CSFRα (*P* = 0.000002). Statistically significant differences in the expression levels of IL-3Rα (*P* < 0.0001), GM-CSFRα (*P* = 0.005), and G-CSFR (*P* < 0.0001) were observed among the cytogenetic risk groups. IL-2Rα (*P* = 0.03) and γc (*P* = 0.04) also showed such a tendency (*P* = 0.03). Of note, only IL-2Rα levels tended to elevate proportionally as cytogenetic risk worsened.

**Table 2:** Correlation of cytokine receptor level with clinicalclinical and cellular features in patients with AML.

	IL-3Rα	GM-CSFRα	IL-2Rα	γc	c-kit	FLT3	G-CSFR
**Age**							
**>60**	**857±139 (91)**	**1427±218 (91)**	**306±71 (229)**	**908±100 (83)** **	**4168±582 (85)**	**1776±274 (33)**	**906±108 (84)** **
**≦60**	**851±130 (163)**	**1397±129 (162)**	**325±53 (389)**	**1358±99 (154)** **	**3256±249 (159)**	**1878±191 (77)**	**1271±124 (160)** **
**Gender**							
**Male**	**816±128 (165)**	**1445±153 (165)**	**325±58 (366)**	**1395±152 (152)**	**3835±375 (156)**	**1686±192 (67)**	**1213±99 (155)**
**Female**	**881±132 (97)**	**1314±149 (96)**	**287±59 (272)**	**1165±136 (92)**	**3139±281 (96)**	**2063±247 (46)**	**1070±176 (97)**
**White blood cell**							
**≧3x10** ^**4**^ **/μL**	**1223±191 (105)** **	**1763±192 (104)** **	**569±106 (204)** **	**1138±149 (98)**	**2410±212 (101)** **	**1819±222 (48)**	**686±63 (99)** **
**<3x10** ^**4**^ **/μL**	**560±90 (139)** **	**1178±144 (139)** **	**177±34 (394)** **	**1383±161 (129)**	**4484±432 (135)** **	**1905±225 (60)**	**1454±147 (136)** **
**Phenotype**							
**CD34+**	**847±128 (174)**	**1141±119 (174)** **	**372±57 (376)** **	**1106±74 (161)**	**4168±357 (168)** **	**1733±163 (88)**	**1191±119 (168)**
**CD34-**	**836±120 (87)**	**1927±226 (86)** **	**219±58 (261)** **	**1700±278 (83)**	**2359±240 (83)** **	**2214±374 (25)**	**1106±134 (83)**
**Karyotype**							
**Favorable**	**422±77 (77)** **	**892±155 (77)** **	**73±17 (162)** *	**1496±143 (72)** *	**4465±508 (77)**	**1822±192 (39)**	**2017±220 (78)** **
**Intermediate**	**1278±179 (122)** **	**1677±167 (122)** **	**354±63 (308)** *	**1232±108 (113)** *	**3008±262 (118)**	**2011±288 (46)**	**818±96 (116)** **
**Unfavorable**	**481±129 (25)** **	**1527±415 (25)**	**613±166 (69)** *	**952±244 (24)**	**4937±1588 (24)**	**1074±198 (15)**	**904±154 (23)** **

Data are given as the mean levels of cytokine receptor expression (sites/cell) ± standard error (number of patients analyzed).

* *p* < 0.05.

**p < 0.01.

### Cytokine receptor expression and clinical outcomes in patients with AML

**Fig 2:**
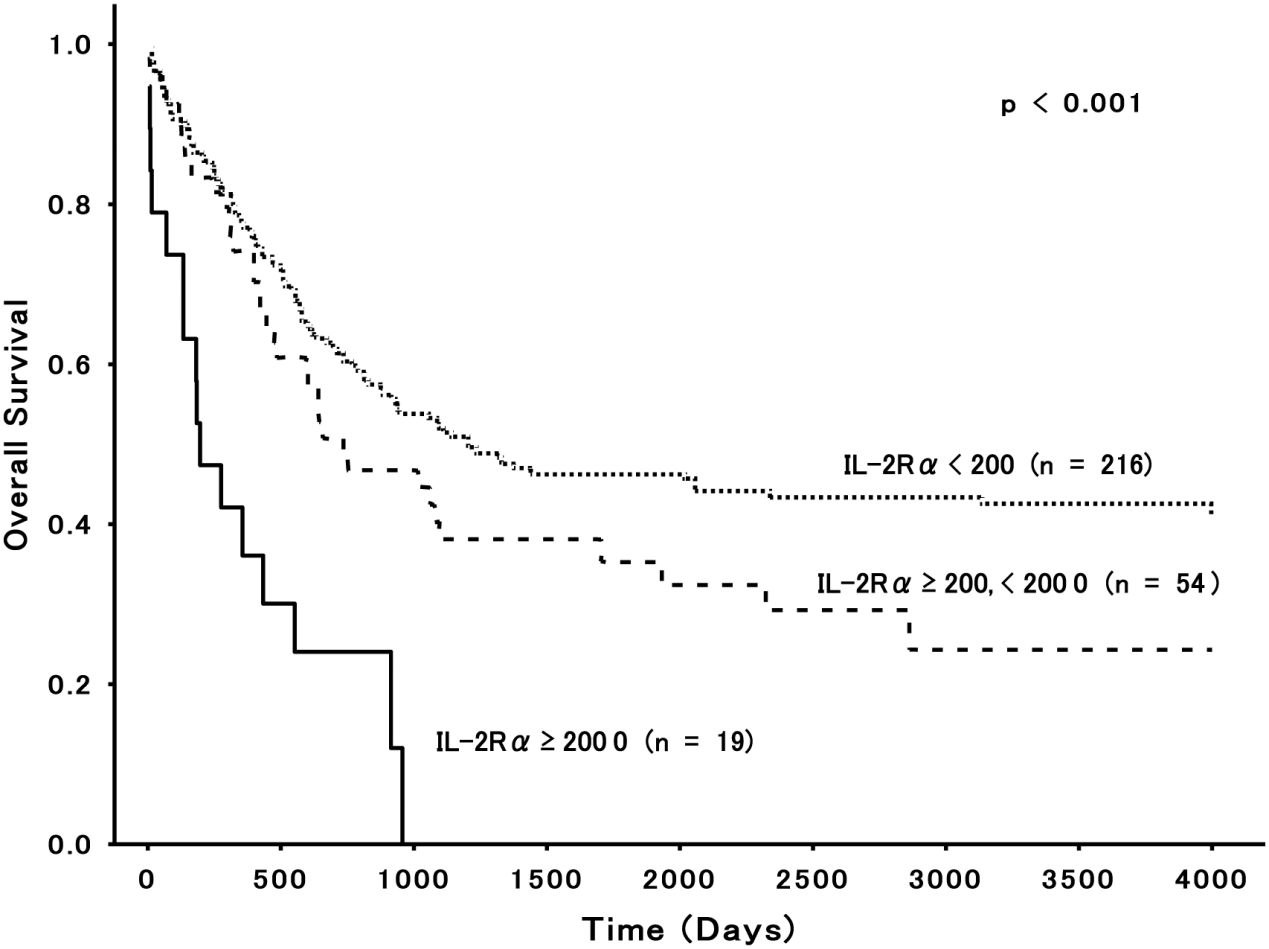
Relationship between IL-2Rα expression levels and OS. Kaplan—Meier estimates of OS for non-M3 patients ≤60 years old are shown according to the expression levels of IL-2Rα (sites/cell).

We correlated clinical outcomes, including CR and OS rates, with cytokine receptor expression levels that showed the wide range (maximum level ≥ 10,000 sites/cell or mean levels ≥ 1,000 sites/cell). To analyze significances, we divided each group into high expressers and low expressers based upon the mean levels of sites/cell. Patients expressing cytokine receptors at levels below or above the mean were classified as low or high expressers, respectively. Table 3 shows the CR and OS rates in patients with non-M3 AML for each group of cytokine receptors, separated by patient age. For patients ≤60 years old, high expressers of IL-3Rα (*P* = 0.007), GM-CSFRα (*P* = 0.001), and IL-2Rα (*P* = 0.002) had lower CR rates than did low expressers of these receptors. Only IL-2Rα correlated with a shorter OS (*P* = 0.001) in high expressers compared with low expressers. The expression of other cytokine receptors including γc, c-kit, FLT3, and G-CSF showed no prognostic relevance. In patients >60 years old, no significant differences were observed in CR and OS rates between high and low expressers of any cytokine receptor. In order to delineate the relationship between IL-2Rα expression levels and OS more clearly, we divided the levels into 3 categories: IL-2Rα < 200; IL-2Rα ≥ 200, but < 2,000; and IL-2Rα ≥ 2,000, and compared OS rates among these groups. OS curves worsened as IL-2Rα levels increased (Fig 2; *P* < 0.001).

**Table 3:** Correlation of cytokine receptor expression with CR rate and OS for patients with non-M3 AML.

Cytokine Receptor	Expression Level	Age ≦ 60 years			Age > 60 years		
		CR rate (%)	P-value	P-value (OS)	CR rate (%)	P-value	P-value (OS)
**IL-3Rα**	**High**	**22/35 (63)**	**0.007**	**0.07**	**10/19 (53)**	**0.60**	**0.72**
	**Low**	**79/92 (86)**			**30/50 (60)**		
**GM-CSFRα**	**High**	**26/45 (58)**	**0.001**	**0.36**	**10/22 (46)**	**0.19**	**0.47**
	**Low**	**75/82 (92)**			**39/47 (64)**		
**βc**	**High**	**40/54 (74)**	**0.26**	**0.57**	**9/22 (41)**	**0.07**	**0.09**
	**Low**	**61/73 (84)**			**31/47 (66)**		
**IL-2Rα**	**High**	**32/54 (59)**	**0.002**	**0.001**	**18/37 (49)**	**0.57**	**0.35**
	**Low**	**188/235 (80)**			**73/134 (55)**		
**γc**	**High**	**35/48 (73)**	**0.11**	**0.61**	**9/14 (64)**	**0.76**	**0.60**
	**Low**	**63/74 (85)**			**28/49 (57)**		
**c-kit**	**High**	**29/39 (74)**	**0.34**	**0.22**	**14/26 (54)**	**0.62**	**0.28**
	**Low**	**70/85 (82)**			**23/38 (61)**		
**FLT3**	**High**	**18/25 (72)**	**0.21**	**0.87**	**18/25 (72)**	**0.48**	**0.53**
	**Low**	**35/41 (85)**			**8/14 (57)**		
**c-fms**	**High**	**20/30 (67)**	**0.01**	**0.72**	**11/17 (65)**	**1.0**	**0.34**
	**Low**	**68/77 (88)**			**23/38 (61)**		
**G-CSFR**	**High**	**33/37 (89)**	**0.14**	**0.32**	**7/13 (54)**	**0.76**	**0.44**
	**Low**	**68/88 (77)**			**29/49 (59)**		
**IL-6Rα**	**High**	**25/35 (71)**	**0.21**	**0.58**	**14/32 (44)**	**0.02**	**0.04**
	**Low**	**72/87 (83)**			**24/33 (73)**		
**GP130**	**High**	**38/46 (83)**	**0.64**	**0.18**	**16/26 (62)**	**0.62**	**0.88**
	**Low**	**59/76 (78)**			**22/40 (55)**		

CR, complete remission; OS, overall survival.

### Effects of cytokines on AML cells

We assessed the relationship between receptor expression levels and responses to each of the cytokines (IL-3, GM-CSF, IL-2, IL-4, M-CSF, G-CSF and IL-6) in leukemia cells from 11 patients with AML (Table 4). The response of AML cells to any cytokine was considered positive when the S.I. value was more than 2. Receptors for several cytokines are composed of multiple chains as follows: IL-3R, IL-3Rα+βc; GM-CSFR, GM-CSFRα+βc; IL-2R, IL-2Rα+IL-2Rβ+γc; IL-4R, IL-4Rα+γc; IL-6R, IL-6Rα+GP130. Of these, βc, IL-2Rβ, γc and GP130 are essential for cytokine signaling. Leukemia cells from most patients were stimulated by IL-3, GM-CSF, or G-CSF regardless of the expression levels of respective receptors. However, there was also a nonresponsive case (case 9) with respect to these cytokines even though this patient’s leukemia cells expressed a considerable number of the corresponding receptors. None of the patients (including those that were high expressers of IL-2Rα and IL-6Rα) responded to IL-2 and IL-6 treatment. IL-4 responsiveness of AML cells showed no correlation with the number of IL-4Rα expressed on their surfaces. The effects of M-CSF tended to depend on the expression levels of c-fms on AML cells.

**Table 4:** Responsiveness of AML cells to cytokines.

Case	IL-3	GM-CSF		IL-2		IL-4		M-CSF	G-CSF	IL-6	
	(IL-3Rα)	(GM-CSFRα)	(βc)	(IL-2Rα)	(IL-2Rβ)	(IL-4Rα)	(γc)	(c-fms)	(G-CSFR)	(IL-6Rα)	(GP130)
**1**	**13.7**	**6.8**		**1.3**		**5.0**		**0.6**	**3.5**	**1.3**	
	**(15243)**	**(5298)**	**(<200)**	**(5298)**	**(<200)**	**(261)**	**(2736)**	**(<200)**	**(<200)**	**(1025)**	**(<200)**
**2**	**2.9**	**3.2**		**1.2**		**1.4**		**1.0**	**1.5**	**0.7**	
	**(540)**	**(680)**	**(200)**	**(<200)**	**(<200)**	**(<200)**	**(901)**	**(<200)**	**(265)**	**(292)**	**(<200)**
**3**	**9.5**	**2.1**		**1.1**		**1.6**		**NT**	**1.5**	**0.2**	
	**(<200)**	**(<200)**	**(254)**	**(<200)**	**(<200)**	**(NT)**	**(632)**	**(<200)**	**(<200)**	**(<200)**	**(<200)**
**4**	**8.3**	**11.3**		**1.7**		**0.6**		**2.4**	**8.1**	**NT**	
	**(4099)**	**(3913)**	**(318)**	**(1769)**	**(<200)**	**(<200)**	**(896)**	**(762)**	**(1074)**	**(318)**	**(260)**
**5**	**6.7**	**6.9**		**1.0**		**0.6**		**2.0**	**3.9**	**NT**	
	**(2645)**	**(2645)**	**(434)**	**(6807)**	**(<200)**	**(<200)**	**(2270)**	**(928)**	**(792)**	**(1401)**	**(<200)**
**6**	**8.8**	**3.5**		**1.1**		**3.7**		**NT**	**2.1**	**0.6**	
	**(<200)**	**(<200)**	**(772)**	**(<200)**	**(<200)**	**(<200)**	**(1247)**	**(<200)**	**(<200)**	**(<200)**	**(<200)**
**7**	**9.2**	**9.0**		**1.1**		**1.1**		**1.5**	**27.9**	**0.6**	
	**(<200)**	**(534)**	**(<200)**	**(<200)**	**(<200)**	**(<200)**	**(<200)**	**(<200)**	**(571)**	**(343)**	**(<200)**
**8**	**25.1**	**19.0**		**1.0**		**NT**		**NT**	**14.7**	**NT**	
	**(815)**	**(439)**	**(609)**	**(3071)**	**(<200)**	**(NT)**	**(587)**	**(<200)**	**(634)**	**(336)**	**(<200)**
**9**	**0.9**	**0.7**		**0.9**		**NT**		**NT**	**1.2**	**NT**	
	**(1889)**	**(6303)**	**(490)**	**(<200)**	**(362)**	**(918)**	**(2533)**	**(6313)**	**(1933)**	**(2491)**	**(588)**
**10**	**4.7**	**4.0**		**1.8**		**NT**		**NT**	**4.5**	**NT**	
	**(751)**	**(6295)**	**(2879)**	**(<200)**	**(347)**	**(<200)**	**(4242)**	**(<200)**	**(6597)**	**(617)**	**(1500)**
**11**	**2.3**	**10.5**		**0.8**		**NT**		**NT**	**3.7**	**NT**	
	**(235)**	**(1071)**	**(<200)**	**(<200)**	**(<200)**	**(<200)**	**(<200)**	**(NT)**	**(738)**	**(287)**	**(<200)**

Data are given as the stimulation index of ^3^H-thymidine uptake (expression levels of cytokine receptors, sites/cell).

NT, not tested.

### Relationship between IL-2Rα expression and expression levels of other cytokine receptors

**Fig 3:**
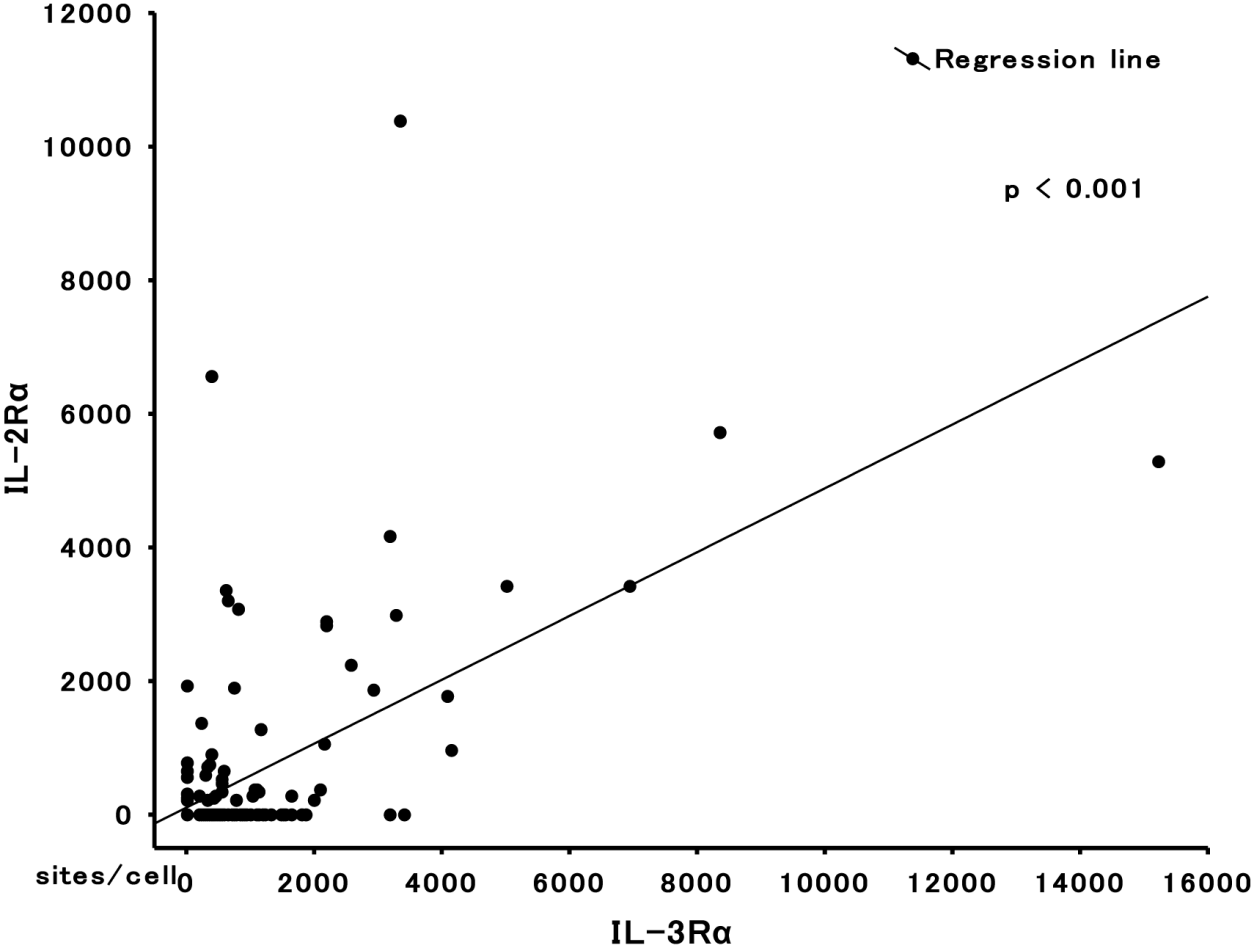
Correlation diagram for the expression levels of IL-2Rα and IL-3Rα. Data are shown for AML patients ≤60 years old.

We evaluated the relationship between the expression levels (sites/cell) of IL-2Rα and those of other cytokine receptors (Table 5). For patients ≤60 years old, expression levels of IL-2Rα correlated with those of IL-3Rα (n = 162, R = 0.59, *P* < 0.001; Fig 3). No statistically significant association was observed between the expression levels of IL-2Rα and those of any other cytokine receptors studied. For patients >60 years old, IL-2Rα expression did not correlate with expression of any other cytokine receptor studied.

**Table 5:** Correlation between the expression levels of IL-2Rα and other cytokine receptors.

Cytokine receptor	Age ≦ 60 years			Age > 60 years		
	No.	R	P-value	No.	R	P-value
**IL-3Rα**	**162**	**0.59**	**< 0.001**	**91**	**0.22**	**0.04**
**GM-CSFRα**	**161**	**0.17**	**0.03**	**91**	**0.13**	**0.23**
**IL-5Rα**	**159**	**-0.09**	**0.26**	**83**	**-0.02**	**0.84**
**βc**	**161**	**-0.05**	**0.49**	**90**	**0.006**	**0.96**
**IL-2Rβ**	**239**	**-0.02**	**0.79**	**133**	**-0.04**	**0.66**
**IL-4Rα**	**144**	**0.06**	**0.47**	**85**	**-0.06**	**0.59**
**IL-7Rα**	**133**	**0.005**	**0.96**	**79**	**-0.04**	**0.71**
**γc**	**154**	**0.06**	**0.49**	**83**	**0.10**	**0.36**
**c-kit**	**158**	**-0.06**	**0.48**	**85**	**-0.07**	**0.50**
**FLT3**	**76**	**0.07**	**0.54**	**33**	**-0.30**	**0.09**
**c-fms**	**136**	**-0.03**	**0.77**	**77**	**0.13**	**0.27**
**c-mpl**	**126**	**0.13**	**0.16**	**71**	**-0.14**	**0.26**
**G-CSFR**	**159**	**-0.14**	**0.07**	**84**	**-0.03**	**0.78**
**IL-6Rα**	**156**	**0.06**	**0.42**	**86**	**0.27**	**0.01**
**GP130**	**156**	**-0.13**	**0.10**	**87**	**-0.06**	**0.59**

No., number of patients examined; R, correlation coefficient.

### Relationship between IL-2Rα expression and phenotypes or karyotypes

**Fig 4:**
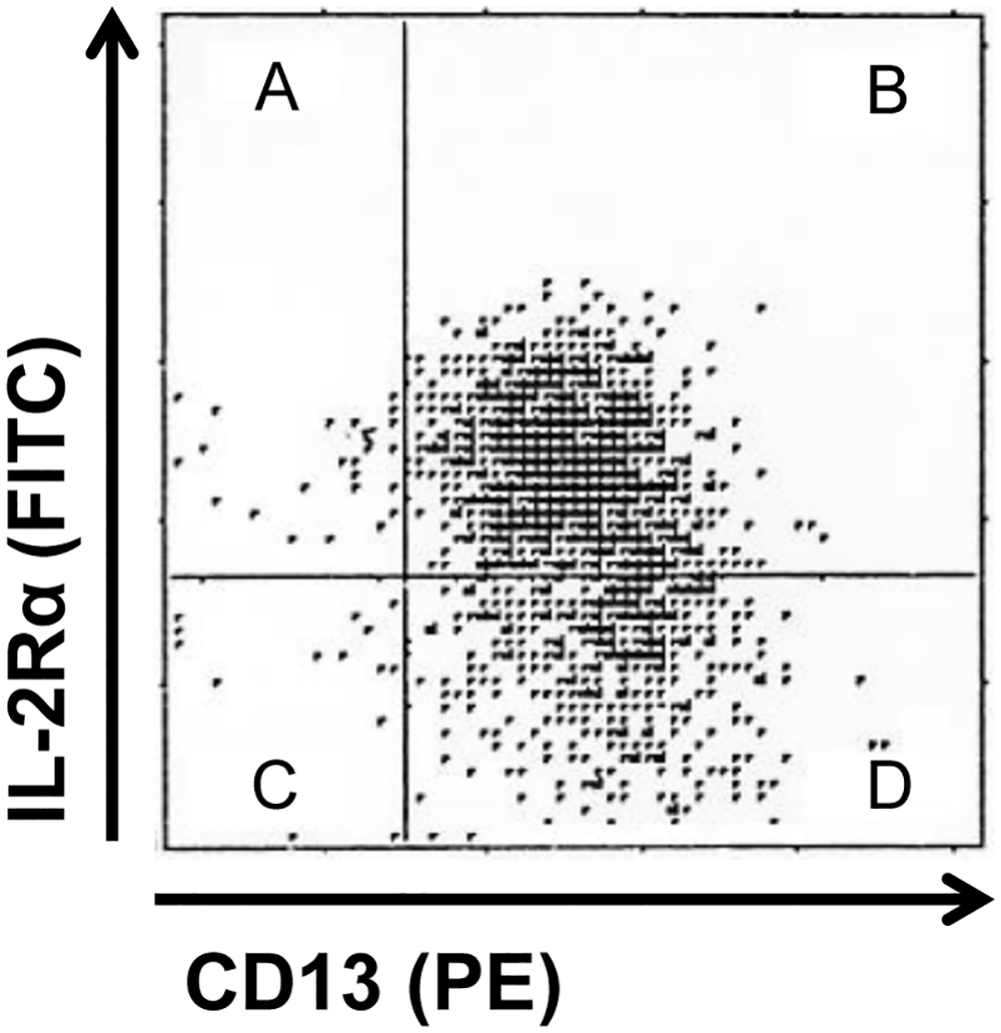
Double immunostaining of leukemia cells using anti-IL-2Rα and anti-CD13 antibodies in a representative patient with IL-2Rα^+^CD4^+^AML. The horizontal axis indicates the logarithmic scale of FITC-conjugated anti- IL-2Rα binding, and the vertical axis PE-conjugated anti-CD13 binding. IL-2Rα and CD13 dual-positive cells are shown in the right upper quadrant (B).

To clarify the cellular characteristics of IL-2Rα^+^ AML, we evaluated the relationship between IL-2Rα expression and various phenotypes or karyotypes in patients with AML (Table 6). To use IL-2Rα as a practical variable, we studied IL-2Rα expression, defined as levels ≥ 309 sites/cell (mean level) or more than 15% cells with immunofluorescence above control cells. For patients ≤60 years old, the expression of IL-2Rα was significantly correlated with that of HLA-DR (*P* < 0.001), CD4 (*P* < 0.001), CD11b (*P* < 0.001), and CD11c (*P* < 0.001). Coexpression of IL-2Rα and myeloid antigen (CD13) on the leukemia cells from a representative case of IL-2Rα^+^CD4^+^AML was confirmed by double immunostaining (Fig 4), showing that IL-2Rα was actually expressed on AML cells, but not on T-lymphocytes. Regarding the karyotype, a significant association was found between IL-2Rα expression and either the absence of t(15; 17) favorable karyotype (*P* < 0.001) or the presence of the t(9; 22) adverse karyotype (*P* = 0.002). However, for patients >60 years old, no significant differences were observed between IL-2Rα expression and the phenotypes or karyotypes studied.

**Table 6:** Correlation of IL-2Rα expression with phenotype and karyotype in patients with AML.

Cellular feature	Age≦60 years			Age>60 years		
	IL-2Rα^+^	IL-2Rα^−^	P-value	IL-2Rα^+^	IL-2Rα^−^	P-value
**Phenotype**						
**HLA-DR**	**676/71 (94)**	**291/390 (75)**	**< 0.001**	**45/50 (90)**	**181/233 (78)**	**0.05**
**HLA-DQ**	**28/64 (44)**	**102/327 (31)**	**0.06**	**14/39 (36)**	**54/197 (27)**	**0.33**
**CD2**	**2/71 (3)**	**44/390 (11)**	**0.03**	**7/50 (14)**	**12/233 (5)**	**0.05**
**CD4**	**34/71 (48)**	**90/390 (23)**	**< 0.001**	**20/50 (40)**	**76/233 (33)**	**0.33**
**CD7**	**23/71 (32)**	**97/390 (25)**	**0.19**	**10/50 (20)**	**44/233 (19)**	**0.84**
**CD10**	**6/71 (8)**	**17/390 (4)**	**0.14**	**1/50 (2)**	**13/233 (6)**	**0.48**
**CD19**	**18/71 (25)**	**64/390 (16)**	**0.09**	**8/50 (16)**	**29/233 (12)**	**0.49**
**CD11b**	**50/71 (70)**	**151/390 (39)**	**< 0.001**	**26/50 (52)**	**90/233 (39)**	**0.06**
**CD11c**	**47/59 (80)**	**139/308 (45)**	**< 0.001**	**27/38 (71)**	**109/193 (57)**	**0.11**
**CD14**	**15/71 (21)**	**75/390 (19)**	**0.75**	**10/50 (20)**	**46/233 (20)**	**1.0**
**CD18**	**48/50 (96)**	**178/210 (85)**	**0.04**	**29/29 (100)**	**112/124 (90)**	**0.12**
**CD56**	**12/50 (24)**	**59/236 (25)**	**1.0**	**11/38 (29)**	**37/150 (25)**	**0.68**
**Karyotype**						
**Normal**	**27/61 (44)**	**128/346 (37)**	**0.32**	**17/43 (40)**	**93/194 (48)**	**0.40**
**t(8;21)**	**8/61 (13)**	**57/347 (16)**	**0.58**	**2/43 (5)**	**17/194 (9)**	**0.54**
**t(15;17)**	**0/61 (0)**	**57/347 (16)**	**< 0.001**	**2/43 (5)**	**16/194 (8)**	**0.54**
**inv(16)/t(16;16)**	**2/61 (3)**	**13/347 (4)**	**1.0**	**0/43 (0)**	**3/193 (2)**	**1.0**
**t(16;21)**	**2/61 (3)**	**0/347 (0)**	**0.02**	**1/43 (2)**	**0/193 (0)**	**0.18**
**t(9;22)**	**4/61 (7)**	**1/346 (0.3)**	**0.002**	**2/43 (5)**	**3/193 (2)**	**0.23**
**t(11q23)**	**2/61 (3)**	**12/347 (3)**	**1.0**	**1/43 (2)**	**3/193 (2)**	**0.53**
**-5/del5(q)**	**0/61 (0)**	**7/347 (2)**	**0.60**	**3/43 (7)**	**11/193 (6)**	**0.72**
**-7/del7(q)**	**2/61 (3)**	**12/347 (3)**	**1.0**	**4/43 (9)**	**12/193 (6)**	**0.50**
**+8**	**3/61 (5)**	**14/347 (4)**	**0.72**	**1/43 (2)**	**13/193 (7)**	**0.48**
**+21**	**0/61 (0)**	**11/347 (3)**	**0.38**	**1/43 (2)**	**4/193 (2)**	**0.96**
**Complex**	**5/61 (8)**	**26/347 (7)**	**0.79**	**5/43 (12)**	**24/193 (12)**	**1.0**

Data are given as number of positive patients/number of patients examined (%).

### Relationship between IL-2Rα expression and cytogenetic risk groups

**Fig 5:**
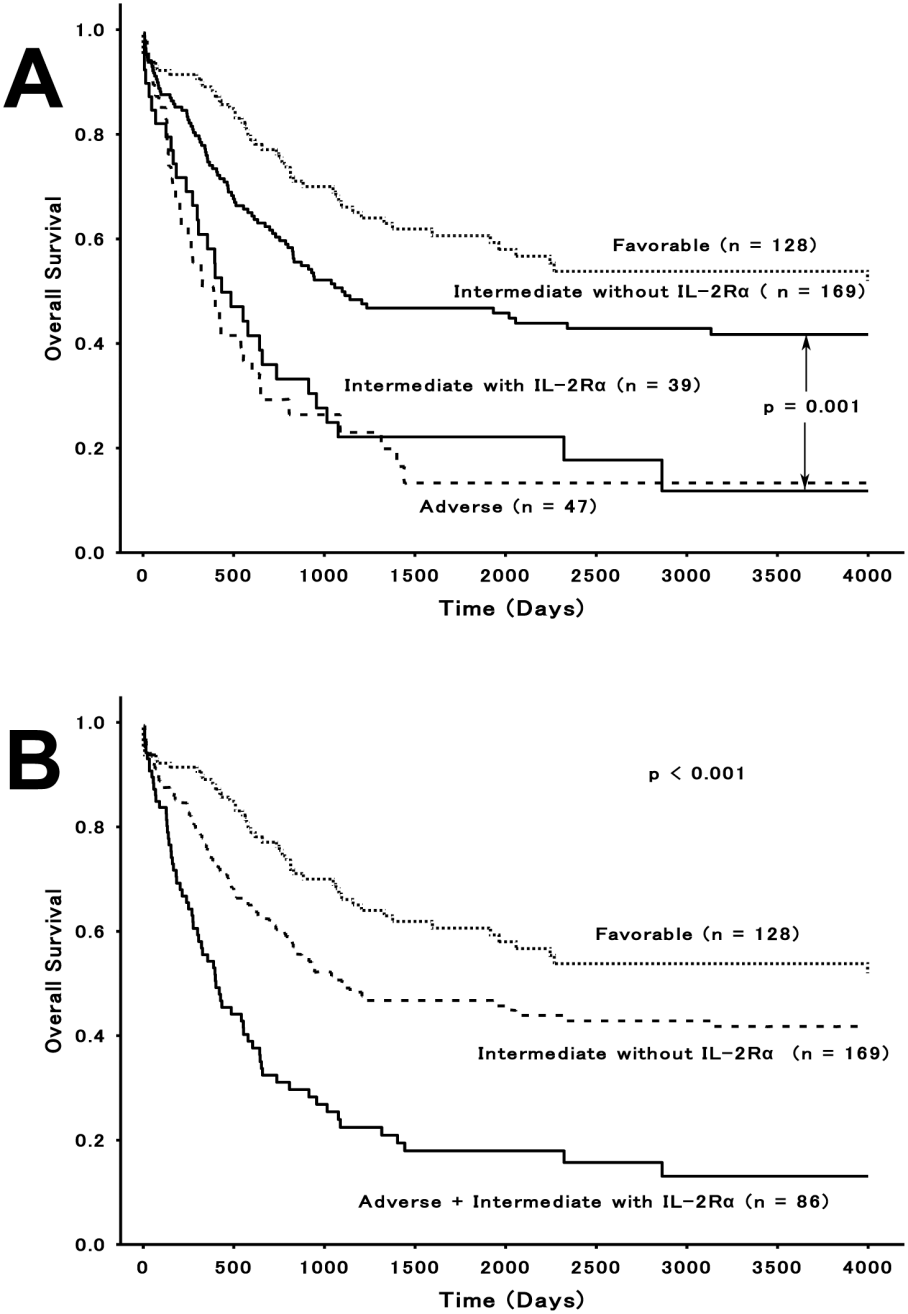
Effect of IL-2Rα expression on cytogenetic-risk classification. Kaplan—Meier estimates of OS for AML patients ≤60 years old are shown based on the 3 distinct cytogenetic-risk groups. (A) The intermediate-risk group was clearly divided into IL-2Rα^+^ patients and IL-2Rα^−^ patients. (B) Revised survival curves show that the original favorable-risk group, the intermediate-risk group in which IL-2Rα^+^ patients were excluded, and the adverse-risk group in which IL-2Rα^+^ patients in the intermediate-risk group were included.

Cytogenetic analysis at the time of diagnosis is the most common basis for determining prognosis in patients with AML. This method is used to stratify risk and identify three discrete risk categories such as the favorable-, intermediate-, and adverse-risk groups. We incorporated the IL-2Rα status into the cytogenetic risk classification scheme and evaluated its prognostic significance. For patients ≤60 years old, IL-2Rα was positive in 8 of 128 (6%) favorable-risk, 39 of 208 (19%) intermediate-risk, and 9 of 47 (19%) adverse-risk patients. In the intermediate-risk group, OS was significantly worse in IL-2Rα^+^ patients (median OS, 431 days; 5-year OS, 22%) than in IL-2Rα^-^ patients (median OS, 1094 days; 5-year OS, 47%; *P* < 0.01; Fig 5A). However, no significant differences in OS rates were revealed between IL-2Rα^+^ and IL-2Rα^−^ patients in the favorable- (*P* = 0.65) and adverse-risk (*P* = 0.21) groups, respectively. However, differences between OS rates in the IL-2Rα^+^ intermediate-risk, the IL-2R^−^ adverse-risk, and the IL-2Rα^+^ adverse-risk groups were not significant (*P* = 0.42). Therefore, we assigned IL-2Rα^+^ intermediate-risk patients to an adverse category. The corresponding OS curves are shown in Fig 5B (*P* < 0.001). For patients >60 years old, IL-2Rα was positive in 3 of 35 (9%) favorable-risk, 24 of 138 (17%) intermediate-risk, and 9 of 36 (25%) adverse-risk patients, respectively. There were no significant differences in OS between IL-2Rα^+^ and IL-2Rα^−^ patients in these risk groups (favorable-risk; *P* = 0.70, intermediate-risk; *P* = 0.26, adverse-risk; *P* = 0.93).

### Relationship between IL-2Rα expression and other prognostic factors in the cytogenetic intermediate-risk group

As IL-2Rα expression showed prognostic value in the cytogenetic intermediate-risk group for patients ≤60 years old, we evaluated the relationship between IL-2Rα expression and other factors such as a WBC count ≥3x10^4^/μl [24], and expression of CD4 [25], CD7 [26], CD11b [27], and CD56 [28] which are recognized as poor prognostic indicators for AML (Table 7). Univariate analyses with the Cox regression method showed that CD4 and IL-2Rα expression correlated with a poor prognosis for OS (*P* = 0.004 and *P* < 0.001, respectively). Multivariate analysis for OS revealed IL-2Rα expression as the only independent adverse factor studied (*P* = 0.002).

**Table 7:** Prognostic factors affecting OS for patients with cytogenetically intermediate-risk (≤60 years).

Prognostic factor	Univariate	Multivariate	
	P-value	Relative risk (99% CI)	P-value
**WBC ≧ 3x10** ^**4**^ **/μl**	**0.03**	**−**	**−**
**CD4**	**0.004**	**1.51 (0.40–5.69)**	**0.04**
**CD7**	**0.35**	**−**	**−**
**CD11b**	**0.14**	**−**	**−**
**CD56**	**0.86**	**−**	**−**
**IL-2Rα(CD25)**	**< 0.001**	**2.17 (1.16–4.08)**	**0.002**

WBC, white blood cell; CI, confidence interval.

## Discussion

In the present study, we demonstrated that all the cytokine receptors evaluated (IL-2Rα, IL-2Rβ, IL-3Rα, IL-4Rα, IL-5Rα, IL-6R, IL-7Rα, βc, γc, GM-CSFRα, G-CSFR, c-fms, c-mpl, c-kit, FLT3, and GP130) were expressed in AML cells with a variable range of expression levels (Table 1). According to the FAB subtype (Fig 1), it is consistent with other reports that GM-CSFRα [29] and c-fms [30] were preferentially expressed in the M4/M5 group, and G-CSFR [31] in the M3 group. Although IL-2Rα was previously shown to be restricted to monocytoid AML (M4/M5) [32], our large series demonstrated its exclusive expression in the non-M3 group, being evenly distributed in both the M1/M2 and the M4/M5 groups.

IL-3Rα, GM-CSFRα, IL-2Rα, γc, c-kit, FLT3, and G-CSFR showed a wide range of expression levels, and all these receptors (except FLT3) showed significant correlations with certain clinical or cellular features of AML (Table 2). The number of WBCs correlated with the expression of IL-3Rα, GM-CSFRα, and IL-2Rα, suggesting a role for these receptors in leukemia progression and treatment outcome. Indeed, high expressers of these receptors showed lower CR rates than did low expressers. In contrast, the levels of c-kit and G-CSFR were negatively associated with WBC counts. Although both SCF [33] and G-CSF [31] are known to stimulate the proliferation of AML cells, these receptors may be mainly involved in cell growth unrelated response at higher expression levels.

Our in vitro observations indicated no correlation between the ability of AML cells to respond to various cytokines and the number of receptors expressed for those cytokines (Table 4). This may be explained by the facts that certain receptors possess defects in their function, and are involved in the activation of cellular functions rather than proliferation [34]. In addition, the levels of receptors are based on average cell estimates, which may not accurately reflect the receptor levels for each cytokine on rare, but biologically crucial clonogenic cells [35]. Thus, the functional significance of cytokine/cytokine receptor systems on AML cells require further investigation.

Clinically, the major finding of this study was that only IL-2Rα status correlated with OS rates of AML patients ≤60 years old. Although results from a previous study [5] indicated that AML patients with elevated IL-3Rα levels had a lower OS rate, our large studies demonstrated no such results. Indeed, for patients ≤60 years old, high expressers of IL-3Rα, GM-CSFRα, and IL-2Rα had similar CR rates (~60%) (Table 3). Unlike high expressers of the former 2 receptors, those of IL-2Rα showed a higher relapse rate and therapy refractoriness even when achieving CR, and finally revealed a significantly poorer OS compared to low expressers. The occurrence of relapse is thought to emerge from the outgrowth of minimal residual disease (MRD) [36]. Accordingly, it is quite intriguing that Terwijn et al [8]. reported that the frequency of MRD was proportional to IL-2Rα expression, and thus this expression was directly related to quality of CR. Furthermore, it is noteworthy that differing from IL-3Rα and GM-CSFRα, a rise of IL-2Rα expression was paralleled linearly by a worsening prognosis (Table 2, Fig 2).

With respect to phenotype, the hematopoietic stem cell (HSC) marker, CD34 was significantly correlated with higher expression levels of IL-2Rα and c-kit, as well as lower levels of GM-CSFRα (Table 2), suggesting that the expression of these receptors depends on the degree of cellular immaturity. However, no association was observed between CD34 and the LSC marker, IL-3Rα. This finding seems to reflect observations that HSCs are heterogeneous, with some being CD34^+^ and some CD34^−^ [37].

The cytogenetic risk classification scheme currently provides the most powerful prognostic information [23]. In this system, however, more than half of AML patients are categorized as the intermediate-risk category, which is considered to be biologically heterogeneous and prognostically further distinguishable [38,39]. By incorporating the IL-2Rα status in this risk analysis, we could sort out a significantly high-risk cohort equivalent to the adverse-risk category from the subset with intermediate-risk cytogenetics (Fig 5A). These Japanese data are consistent with data found in published reports from western countries [8,9], revealing no ethnic difference [40] in the prognostic effect of IL-2Rα expression. In the intermediate-risk group, IL-2Rα expression was also identified as sole independent factor for OS in our analyses which included other prognostic indicators such as leukocytosis [24], and the expression of well-recognized prognostic surface markers (Table 7) [25–28]. These results also support the prognostic value of IL-2Rα expression in this subgroup of AML.

Molecular markers such as the tandem duplication of FLT3, and mutations in DNMT3A and NPM1 [41–43] have also been reported as factors stratifying the cytogenetically intermediate-risk AML group. Although we could not perform genetic analyses, Gonen, et al [9]. recently described that IL-2Rα expression impacted clinical outcome independent of the presence of these prognostic mutations. Because molecular insights have not become manifested in a routine clinical practice, and are not available for all patients, we recommend that IL-2Rα testing, which is cost-effective and less time-consuming, be combined with cytogenetic risk evaluation and that IL-2Rα^+^ intermediate-risk patients are assigned to an adverse-risk group to improve AML prognostication in a shorter time (Fig 5B).

The biological relevance of IL-2Rα expression is still unclear [44,45]. In agreement with previous studies [46], the extremely low expression level of IL-2Rβ (34 sites/cell) (Table 1), which is essential for IL-2 signaling [47], clearly seems to indicate the lack of IL-2 responsiveness of AML cells (Table 4) [48,49]. This observation allows us to speculate that IL-2Rα serves a role distinct from that of the other growth factor receptors. Of note, a close relationship existed between the expression of IL-2Rα and that of CD4, CD11b, CD11c, and HLA-DR (Table 6), as well as IL-3Rα (Table 5, Fig 3). These results raise the possibility that IL-2Rα^+^AML cells may have not only a reportedly LSC nature [8,9,13], but also a dendritic cell like phenotype suitable for cell-to-cell interactions [49–51]. Data from some reports have demonstrated a marked increase of serum soluble IL-2Rα (sIL-2R) in IL-2Rα^+^ AML cells [52,53]. An active biological role of sIL-2R has been described in lymphoma [54] wherein sIL-2R binds IL-2 and the sIL-2R-IL-2 complex promotes environmental T cell differentiation into regulatory T-cells. Therefore, a similar situation, which could lead to anti-leukemia immune escape status, may be generated in the BM microenvironment of IL-2Rα^+^ AML cells. Since this issue may be fundamentally important for understanding the pathogenesis of the dismal clinical course of IL-2Rα^+^ AML, further investigations are expected.

Findings in this study also demonstrated that a clinical relevance of cytokine receptor expression was observed in patients ≤60 years old, but not in patients older than 60 (Table 3). In addition, the levels of receptors such as γc and G-CSFR were diminished in elderly patients (Table 2). HSCs and BM microenvironments appear to vary significantly with aging, and those changes potentially influence the nature of leukemia cells [55]. Recently, age-related epigenetic drift has also been clarified [56]. Thus, further detailed works are required to better understand the relationship between aging and the biology of AML cells.

In conclusion, several cytokine receptors were associated with certain cellular and clinical features, but only IL-2Rα had prognostic value, which provides an additional marker for better current-risk evaluation in AML patients ≤60 years old. At present, since even allogeneic HSC transplantation showed a limited effect for IL-2Rα^+^ AML [57], newer therapeutic approaches targeting IL-2Rα are expected to improve the prognosis of this type of AML.
